# Synthesis of Methacrylate Monomers with Antibacterial Effects Against *S. Mutans*

**DOI:** 10.3390/molecules16119755

**Published:** 2011-11-23

**Authors:** Jingwei He, Eva Söderling, Monica Österblad, Pekka K. Vallittu, Lippo V. J. Lassila

**Affiliations:** 1 Department of Biomaterials Science, Institute of Dentistry and Biocity Turku Biomaterial Research Program, University of Turku, Turku 20520, Finland; 2 Turku Clinical Biomaterials Centre-TCBC, University of Turku, Turku 20520, Finland; 3 College of Materials Science and Engineering, South China University of Technology, Guangzhou 510641, China; 4 Institute of Dentistry, University of Turku, Turku 20520, Finland; 5 Antimicrobial Resistance Unit, National Institute for Health and Welfare, Turku 20520, Finland

**Keywords:** dental materials, methacrylate, quaternary ammonium compounds, antibacterial

## Abstract

A series of polymerizable quaternary ammonium compounds were synthesized with the aim of using them as immobilized antibacterial agents in methacrylate dental composites, and their structures were characterized by FT-IR, ^1^H-NMR, and ^13^C-NMR analysis. Their antibacterial activities against the oral bacterium *Streptococcus mutans* were evaluated *in vitro* by a Minimum Inhibitory Concentration test, and the results showed that 2-dimethyl-2-hexadecyl-1-methacryloxyethyl ammonium iodide (**C16**) had the highest antibacterial activity against *S. mutans*, and 2-dimethyl-2-pentyl-1-methacryloxyethyl ammonium iodide (**C5**) and 2-dimethyl-2-octyl-1-methacryloxyethyl ammonium iodide (**C8**) did not show any inhibition.

## 1. Introduction

Dental composite materials which consist of methacrylate monomers and inorganic fillers are widely used in clinic because of their aesthetic superiority and strong bonding ability to tooth substances. However, because they have no intrinsic antibacterial activity, dental composite materials have already been reported as accumulating more plaque than other restorative materials such as ceramics and metals *in vitro* [1,2,3,4] or *in vivo* [5,6,7,8]. Plaque accumulation adjacent to the restoration margins may lead to secondary caries *in vivo* and shorten the life of composite restoration [9]. The more dental plaque accumulates on dental composite materials, the greater the risk of occurrence of caries. Therefore, antibacterial activity is an important property of dental composite materials for successful restoration. 

Quaternary ammonium compounds are well known and effective antibacterial agents, and are used in many fields, such as water treatment, medicine and healthcare products, food applications, and textile products [10,11,12]. Polymerizable quaternary ammonium compounds can polymerize into the polymer network and immobilize the antibacterial agents in polymer backbone to afford the polymer with long-term antibacterial effectiveness. The example of polymerizable quaternary ammonium compounds used as antibacterial monomer in dental restorative materials is methacryloyloxydodecyl pyrimidinium bromide (MDPB), which was prepared by Imazato and co-workers [13,14,15,16,17]. It was reported that bactericide-immobilized dental composite by adding MDPB showed bactericidal activity against *Streptococcus mutans* for a long time. Moreover, MDPB has no influence on mechanical properties of dental composite [13]. 

The objective of this study was to synthesize a series of methacrylate monomers containing quaternary ammonium with different length of alkyl chains, and discuss the relationship between the length of alkyl chain and antibacterial activity. These methacrylate monomers may be used in the future dental composite materials as immobilized antibacterial agents.

## 2. Results and Discussion

### 2.1. Synthesis

**Scheme 1:**
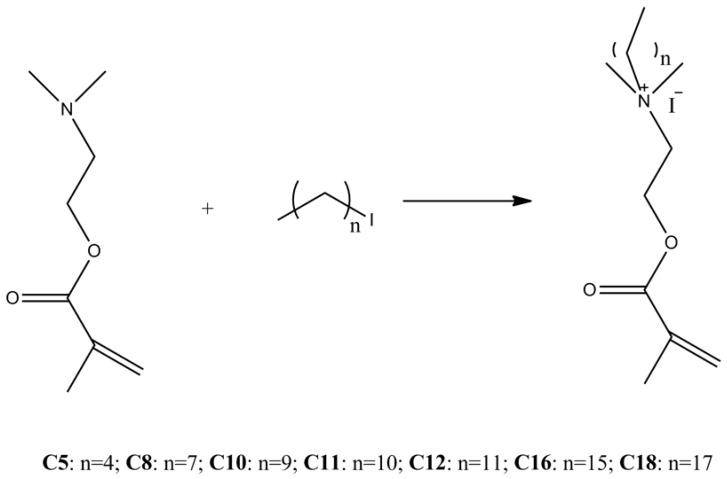
Synthetic pathway for methacrylate monomers containing quaternary ammonium ions.

The polymerizable quaternary ammonium compounds were formed by reacting the polymerizable amine with different commercial alkyl iodides through a Menschutkin reaction, as shown in Scheme 1. The reaction was conducted under solvent-free conditions, to give the products in good yields and purity. The structures of all products were confirmed by their FT-IR, ^1^H-NMR and ^13^C-NMR spectra. In the ^1^H-NMR and ^13^C-NMR spectra, distinctive signals assigned to N^+^CH_2_CH_2_(CH_2_)_n_CH_3_ (around 3.57–3.66 pm) and N^+^CH_2_CH_2_(CH_2_)_n_CH_3_ (65.8 pm) were observed, which means the alkyl iodides have already reacted with dimethylaminoethyl methacrylate (DMAEMA) to form the quaternary ammonium structure. Absorption peaks around 1720 cm^−^^1^ and 1636 cm^−^^1^ in FT-IR spectra, chemical shifts around 6.11–6.17 (CH_2_=C(CH_3_) *trans*) and 5.58–5.64 (CH_2_=C(CH_3_) *cis*) in the ^1^H-NMR spectra, and chemical shifts around 127.4–127.6 (OC(O)C(CH_3_)=CH_2_) in the ^13^C-NMR spectra revealed that there were still methacrylate groups in the products. Above all, the structures of products were as designed.

### 2.2. Antibacterial Activity-MIC

Evaluation of the antibacterial properties of the synthesized methacrylate monomers in this study was conducted using MIC (Minimum Inhibitory Concentration) measurement *vs.* *Streptococcus mutans *Ingbritt, which is a caries-associated bacterium of dental plaque [18]. MIC results are shown in Table 1. As shown in the Table, **C16** (2.5 µg/mL) was the best inhibitor in all synthesized monomers, while **C10** (25 µg/mL), **C11** (6.25 µg/mL), **C12** (6.25 µg/mL), and **C18** (5.0 µg/mL) showed some inhibition, **C5** and **C8** had no inhibition, and all of the synthesized monomers showed less inhibition than the well-known broad-spectrum antimicrobial agent chlorhexidine, which has MIC-values towards *S. mutans* strains ranging from 0.25–1 µg/mL [19]. These results showed that all of the synthesized monomers had antibacterial activity, except **C5** and **C8**, and the alkyl chain length of the monomer had a significant effect on its antibacterial activity.

**Table 1:** MIC results of synthesized monomers and chlorhexidine.

Compound	MIC (µg/mL)
Chlorhexidine	1
**C5**	—
**C8**	—
**C10**	25
**C11**	6.25
**C12**	6.25
**C16**	2.5
**C18**	5

Generally, it has been reported that there are three mechanisms by which polymeric quaternary ammonium compounds may kill bacteria: (1) adsorption onto the negatively charged bacterial cell surface; (2) diffusion through the cell wall and binding to the cytoplasmic membrane; and (3) disruption of the cytoplasmic membrane, release of cytoplasmic constituents and cell death [20,21,22]. It has also been found that the length of the substituent alkyl chain is one of the keys to the antibacterial ability of quaternary ammonium compounds: the longer the substituent alkyl chain, the higher the antibacterial activity [20,21,23]. However, in this research, the antibacterial activity of synthesized monomer increased when the length of substituent alkyl chain increased from 5 to 16, and decreased when the length of the alkyl chain increased further to 18. This phenomenon is a typical cut-off effect which has already been observed in various biological and toxic activities of long-chain surface-active substances [24]. Many assumptions have been proposed to explain the origin of the cut-off effect, among them, the concept of free volume could be applied to quaternary ammonium salts. In solution, the polar ammonium heads interact with polar groups of the phospholipids of the bacterial and their hydrocarbon chains will orient parallel to the hydrocarbon chains of phospholipids. In this location, the density of the bilayer hydrophobic region must be influenced and a free volume is formed. If the hydrocarbon chain of the ammonium salts is shorter than that of phospholipids, the free volume created in the bilayer hydrophobic region will be small. When the length of hydrocarbon chain of the ammonium salts becomes comparable to that of phospholipids, the free volume decreases and tends towards zero. Ammonium salts with chains between these extrema will induce maximal free volume in the bilayer. The larger the free volume, the more the membrane of bacteria is expect to be destabilized and the bactericidal activity increase [24,25]. Therefore, in this research, the highest antibacterial activity of **C16** might be attributed to the largest free volume in bilayer induced by the hydrocarbon chain of **C16**.

Synthesized monomers from **C10** to **C18** showed significant antibacterial activity, and they could be used as immobilized antibacterial agents in methacrylate dental composites. Further studies should be done to explore the antibacterial activity of dental composites with these synthesized monomers, and the influences of these monomers on physiochemical properties of relevant dental composites also need to be investigated.

## 3. Experimental

### 3.1. Materials and Reagents

All solvents and reagents were purchased from Sigma-Aldrich Co. with high purity, and were used without further purification. FT-IR spectra were obtained on a Spectrum One Fourier Transform Infrared spectrometer (Perkin Elmer Inc.). ^1^H-NMR and ^13^C-NMR spectra were measured in CDCl_3_ solution on a LK 500 MHz spectrometer (Bruker Co.). 

### 3.2. General Procedure for the Synthesis of Methacrylate Quaternary Ammoniums ***C5**-**C18***

Dimethylaminoethyl methacrylate (DMAEMA, 0.06 mol) was reacted with alkyl iodide (0.05 mol) under solvent-free conditions at a temperature of 50 °C and in the presence of 0.05% of hydroquinone. After 12 h reaction, the solid product was filtered and washed with diethyl ether for several times. Then the white quaternary ammonium compounds were dried under vacuum at 40 °C for 48 h. 

*2-Dimethyl-2-pentyl-1-methacryloxyethyl ammonium iodine *(**C5**). Yield: 75%. FT-IR: ν(cm^−1^) 2968, 2953, 2938, 2873, 1720, 1637, 1467, 1455, 1321, 1297, 1161. ^1^H-NMR (δ ppm): 6.11 (1H, CH_2_=C(CH_3_) *trans*, s), 5.64 (1H, CH_2_=C(CH_3_) *cis*, s), 4.62 (2H, N^+^CH_2_CH_2_OC(O), t), 4.09 (2H, N^+^CH_2_CH_2_OC(O), t), 3.62–3.66 (2H, N^+^CH_2_CH_2_(CH_2_)_2_CH_3_, t), 3.44 (6H, N^+^(CH_3_)_2_, s), 1.91 (3H, CH_2_=C(CH_3_), s), 1.76 (2H, N^+^CH_2_CH_2_(CH_2_)_2_CH_3_, m), 1.33–1.36 (4H, N^+^CH_2_CH_2_(CH_2_)_2_CH_3_, m), 0.85–0.88 (3H, N^+^CH_2_CH_2_(CH_2_)_2_CH_3_, t). ^13^C-NMR (δ ppm): 166.3 (OC(O)-C(CH_3_)=CH_2_), 135.1 (OC(O)C(CH_3_)=CH_2_), 127.5 (OC(O)C(CH_3_)=CH_2_), 65.8 (N^+^CH_2_CH_2_(CH_2_)_2_CH_3_), 62.5 (N^+^CH_2_CH_2_OC(O)), 58.2 (N^+^CH_2_CH_2_OC(O)), 52.2 (N^+^(CH_3_)_2_), 28.1 (N^+^CH_2_CH_2_CH_2_CH_2_CH_3_), 22.6 (N^+^CH_2_CH_2_CH_2_CH_2_CH_3_), 22.3 (N^+^CH_2_CH_2_CH_2_CH_2_CH_3_), 18.3 (CH_2_=C(CH_3_)), 13.9 (N^+^CH_2_CH_2_-CH_2_CH_2_CH_3_). 

*2-Dimethyl-2-octyl-1-methacryloxyethyl ammonium iodine* (**C8**). Yield: 72%. FT-IR: ν(cm^−1^) 2989, 2923, 2855, 1717, 1635, 1467, 1452, 1319, 1296, 1158. ^1^H-NMR (δ ppm): 6.06 (1H, CH_2_=C(CH_3_) *trans*, s), 5.58 (1H, CH_2_=C(CH_3_) *cis*, s), 4.58 (2H, N^+^CH_2_CH_2_OC(O), t), 4.04 (2H, N^+^CH_2_CH_2_OC(O), t), 3.57–3.60 (2H, N^+^CH_2_CH_2_(CH_2_)_5_CH_3_, t), 3.39 (6H, N^+^(CH_3_)_2_, s), 1.85 (3H, CH_2_=C(CH_3_), s), 1.70 (2H, N^+^CH_2_CH_2_(CH_2_)_5_CH_3_, m), 1.16–1.26 (10H, N^+^CH_2_CH_2_(CH_2_)_5_CH_3_, m), 0.76–0.78 (3H, N^+^CH_2_CH_2_(CH_2_)_5_CH_3_, t). ^13^C-NMR (δ ppm): 166.2 (OC(O)-C(CH_3_)=CH_2_), 135.0 (OC(O)C(CH_3_)=CH_2_), 127.4 (OC(O)C(CH_3_)=CH_2_), 65.7 (N^+^CH_2_CH_2_(CH_2_)_5_CH_3_), 62.5 (N^+^CH_2_CH_2_OC(O)), 58.1 (N^+^CH_2_CH_2_OC(O)), 52.2 (N^+^(CH_3_)_2_), 31.5 (N^+^CH_2_CH_2_CH_2_-(CH_2_)_2_HH_2_CH_2_CH_3_), 29.0 (N^+^CH_2_CH2CH2(CH_2_)_2_CH_2_CH_2_CH_3_), 26.0 (N^+^CH_2_CH_2_CH_2_(CH_2_)_2_-CH_2_CH_2_CH_3_), 22.9 (N^+^CH_2_CH_2_CH_2_(CH_2_)_2_CH_2_CH_2_CH_3_), 22.4 (N^+^CH_2_CH_2_CH_2_(CH_2_)_2_-CH_2_CH_2_CH_3_), 18.3(CH_2_=C(CH_3_)), 14.0 (N^+^CH_2_CH_2_CH_2_(CH_2_)_2_CH_2_CH_2_CH_3_).

*2-Dimethyl-2-octyl-1-methacryloxyethyl ammonium iodine* (**C10**). Yield: 81%. FT-IR: ν(cm^−1^) 2957, 2919, 2854, 1716, 1636, 1466, 1453, 1319, 1295, 1156. ^1^H-NMR (δ ppm): 6.16 (1H, CH_2_=C(CH_3_)*trans*, s), 5.68 (1H, CH_2_=C(CH_3_) *cis*, s), 4.67 (2H, N^+^CH_2_CH_2_OC(O), t), 4.14 (2H, N^+^CH_2_CH_2_OC(O), t), 3.63–3.66 (2H, N^+^CH_2_CH_2_(CH_2_)_7_CH_3_, t), 3.50 (6H, N^+^(CH_3_)_2_, s), 1.95 (3H, CH_2_=C(CH_3_), s), 1.76–1.79 (2H, N^+^CH_2_CH_2_(CH_2_)_7_CH_3_, m), 1.25–1.35 (14H, N^+^CH_2_CH_2_(CH_2_)_7_CH_3_, m), 0.86–0.89 (3H, N^+^CH_2_CH_2_(CH_2_)_7_CH_3_, t). ^13^C-NMR (δ ppm): 166.3 (OC(O)C(CH_3_)=CH_2_), 135.1 (OC(O)C(CH_3_)=CH_2_), 127.6 (OC(O)C(CH_3_)=CH_2_), 65.8 (N^+^CH_2_CH_2_(CH_2_)_7_CH_3_), 62.5 (N^+^CH_2_CH_2_OC(O)), 58.1 (N^+^CH_2_CH_2_OC(O)), 52.2 (N^+^(CH_3_)_2_), 31.8 (N^+^CH_2_CH_2_CH_2_(CH_2_)_4_CH_2_CH_2_CH_3_), 29.2–29.4 (N^+^CH_2_CH_2_CH_2_(CH_2_)_4_CH_2_CH_2_CH_3_), 26.2 (N^+^CH_2_CH_2_CH_2_(CH_2_)_4_CH_2_CH_2_CH_3_), 23.0 (N^+^CH_2_CH_2_CH_2 _ (CH_2_)_4_CH_2_CH_2_CH_3_), 22.6 (N^+^CH_2_–CH_2_CH_2_(CH_2_)_4_CH_2_CH_2_CH_3_), 18.3 (CH_2_=C(CH_3_)), 14.1 (N^+^CH_2_CH_2_CH_2_(CH_2_)_4_CH_2_CH_2_CH_3_).

*2-Dimethyl-2-decyl-1-methacryloxyethyl ammonium iodine* (**C11**). Yield: 77%. FT-IR: ν(cm^−1^) 2954, 2921, 2854, 1717, 1636, 1466, 1425, 1319, 1157. ^1^H-NMR (δ ppm): 6.17 (1H, CH_2_=C(CH_3_) *trans*, s), 5.69 (1H, CH_2_=C(CH_3_) *cis*, s), 4.67 (2H, N^+^CH_2_CH_2_OC(O), t), 4.15 (2H, N^+^CH_2_CH_2_OC(O), t), 3.63–3.66 (2H, N^+^CH_2_CH_2_(CH_2_)_8_CH_3_, t), 3.51 (6H, N^+^(CH_3_)_2_, s), 1.96 (3H, CH_2_=C(CH_3_), s), 1.79 (2H, N^+^CH_2_CH_2_(CH_2_)_8_CH_3_, m), 1.26–1.36 (16H, N^+^CH_2_CH_2_(CH_2_)_8_CH_3_, m), 0.87–0.89 (3H, N^+^CH_2 _CH_2_(CH_2_)_8_CH_3_, t). ^13^C-NMR (δ ppm): 166.3 (OC(O)C-(CH_3_)=CH_2_), 135.1 (OC(O)C(CH_3_)=CH_2_), 127.6 (OC(O)C(CH_3_)=CH_2_), 65.8 (N^+^CH_2_-CH_2_(CH_2_)_8_CH_3_), 62.5(N^+^CH_2_CH_2_OC(O)), 58.1 (N^+^CH_2_CH_2_OC(O)), 52.2 (N^+^(CH_3_)_2_), 31.9 (N^+^CH_2_CH_2_CH_2_-(CH_2_)_5_CH_2_CH_2_CH_3_), 29.2–29.5 (N^+^CH_2_CH_2_CH_2_(CH_2_)_5_CH_2_CH_2_CH_3_), 26.2 (N^+^CH_2_CH_2_CH_2_(CH_2_)_5_-CH_2_CH_2_CH_3_), 23.0 (N^+^CH_2_CH_2_CH_2_(CH_2_)_5_CH_2_CH_2_CH_3_), 22.7 (N^+^CH_2_CH_2_CH_2_(CH_2_)_5_CH_2_CH_2_CH_3_), 18.3 (CH_2_=C(CH_3_)), 14.1 (N^+^CH_2_CH_2_CH_2 _(CH_2_)_5_CH_2_CH_2_CH_3_).

*2-Dimethyl-2-dodecyl-1-methacryloxyethyl ammonium iodine* (**C12**). Yield: 85%. FT-IR: ν(cm^−1^) 2955, 2916, 2850, 1718, 1632, 1465, 1453, 1320, 1297, 1163. ^1^H-NMR (δ ppm): 6.14 (1H, CH_2_=C(CH_3_) *trans*, s), 5.66 (1H, CH_2_=C(CH_3_) *cis*, s), 4.64–4.66 (2H, N^+^CH_2_CH_2_OC(O), t), 4.12–4.14 (2H, N^+^CH_2_CH_2_-OC(O), t), 3.62–3.66 (2H, N^+^CH_2_CH_2_(CH_2_)_9_CH_3_, t), 3.49 (6H, N^+^(CH_3_)_2_, s), 1.94 (3H, CH_2_=C(CH_3_), s), 1.75–1.77 (2H, N^+^CH_2_CH_2_(CH_2_)_9_CH_3_, m), 1.23–1.34 (18H, N^+^CH_2_-CH_2_(CH_2_)_9_CH_3_, m), 0.85–0.87 (3H, N^+^CH_2_CH_2_(CH_2_)_9_CH_3_, t). ^13^C-NMR (δ ppm): 166.3 (OC(O)C(CH_3_)=CH_2_), 135.1 (OC(O)C(CH_3_)=CH_2_), 127.5 (OC(O)C(CH_3_)=CH_2_), 65.8 (N^+^CH_2_CH_2 _(CH_2_)_9_CH_3_), 62.5 (N^+^CH_2_CH_2_OC(O)), 58.1 (N^+^CH_2_CH_2_OC(O)), 52.2 (N^+^(CH_3_)_2_), 31.9 (N^+^CH_2_CH_2_CH_2_(CH_2_)_6_CH_2_CH_2_CH_3_), 29.2–29.6 (N^+^CH_2_CH_2_CH_2_(CH_2_)_6_CH_2_CH_2_CH_3_), 26.2 (N^+^–CH_2_CH_2_CH_2_(CH_2_)_6_CH_2_CH_2_CH_3_), 23.0 (N^+^CH_2_CH_2_CH_2_(CH_2_)_6_CH_2_CH_2_CH_3_), 22.6 (N^+^CH_2_CH_2_CH_2_(CH_2_)_6_CH_2_CH_2_CH_3_), 18.3 (CH_2_=C(CH_3_)), 14.1 (N^+^CH_2_CH_2_CH_2_(CH_2_)_6_CH_2_CH_2_CH_3_).

*2-Dimethyl-2-hexadecyl-1-methacryloxyethyl ammonium iodine* (**C16**). Yield: 83%. FT-IR: ν(cm^−^^1^) 2945, 2915, 2849, 1719, 1632, 1465, 1453, 1321, 1297, 1165. ^1^H-NMR (δ ppm): 6.12 (1H, CH_2_=C(CH_3_) *trans*, s), 5.64 (1H, CH_2_=C(CH_3_) *cis*, s), 4.62–4.64 (2H, N^+^CH_2_CH_2_OC(O), t), 4.09–4.11 (2H, N^+^CH_2_CH_2_OC(O), t), 3.61–3.64 (2H, N^+^CH_2_CH_2_(CH_2_)_13_CH_3_, t), 3.46 (6H, N^+^(CH_3_)_2_, s), 1.91 (3H, CH_2_=C(CH_3_), s), 1.73–1.76 (2H, N^+^CH_2_CH_2_(CH_2_)_13_CH_3_, m), 1.21–1.31 (26H, N^+^CH_2_CH_2_(CH_2_)_13_CH_3_, m), 0.82–0.85 (3H, N^+^CH_2_CH_2_(CH_2_)_13_CH_3_, t). ^13^C-NMR (δ ppm): 166.3 (OC(O)C(CH_3_)=CH_2_), 135.1 (OC(O)C(CH_3_)=CH_2_), 127.5 (O-C(O)C(CH_3_)=CH_2_), 65.8 (N^+^CH_2_CH_2_(CH_2_)_13_CH_3_), 62.5 (N^+^CH_2_CH_2_OC(O)), 58.1 (N^+^CH_2_CH_2_OC(O)), 52.2 (N^+^(CH_3_)_2_), 31.9 (N^+^CH_2_CH_2_CH_2_(CH_2_)_10_CH_2_CH_2_CH_3_), 29.2–29.6 (N^+^CH_2_CH_2_CH_2_(CH_2_)_10_CH_2_CH_2_CH_3_), 26.2 (N^+^-CH_2_CH_2_CH_2_(CH_2_)_10_CH_2_CH_2_CH_3_), 22.9 (N^+^CH_2_CH_2_CH_2_(CH_2_)_10_CH_2_CH_2_CH_3_), 22.6 (N^+^CH_2_CH_2_CH_2_(CH_2_)_10_CH_2_CH_2_CH_3_), 18.3 (CH_2_=C(CH_3_)), 14.1 (N^+^CH_2_CH_2_CH_2_(CH_2_)_10_CH_2_CH_2_CH_3_). 

*2-Dimethyl-2-octadectyl-1-methacryloxyethyl ammonium iodine* (**C18**). Yield: 72%. FT-IR: ν(cm^−^^1^) 2944, 2915, 2849, 1719, 1632, 1464, 1453, 1321, 1297, 1166. ^1^H-NMR (δ ppm): 6.14 (1H, CH_2_=C(CH_3_) *trans*, s), 5.65 (1H, CH_2_=C(CH_3_) *cis*, s), 4.64–4.65 (2H, N^+^CH_2_CH_2_OC(O), t), 4.12–4.13 (2H, N^+^CH_2_CH_2_-OC(O), t), 3.62–3.65 (2H, N^+^CH_2_CH_2_(CH_2_)_15_CH_3_, t), 3.48 (6H, N^+^(CH_3_)_2_, s), 1.93 (3H, CH_2_=C(CH_3_), s), 1.76 (2H, N^+^CH_2_CH_2_(CH_2_)_15_CH_3_, m), 1.23–1.33 (30H, N^+^CH_2_CH_2_-(CH_2_)_15_CH_3_, m), 0.84–0.86 (3H, N^+^CH_2_CH_2_(CH_2_)_15_CH_3_, t). ^13^C-NMR (δ ppm): 166.3 (OC(O)C(CH_3_)=CH_2_), 135.1 (OC(O)C(CH_3_)=CH_2_), 127.5 (O–C(O)C(CH_3_)=CH_2_), 65.8 (N^+^CH_2_CH_2 _(CH_2_)_15_CH_3_), 62.5 (N^+^CH_2_CH_2_OC(O)), 58.1 (N^+^CH_2_CH_2_OC(O)), 52.2 (N^+^(CH_3_)_2_), 31.9 (N^+^CH_2_CH_2_ CH_2_(CH_2_)_12_CH_2_CH_2_CH_3_), 29.2–29.7 (N^+^CH_2_CH_2_CH_2_(CH_2_)_12_CH_2_CH_2_CH_3_), 26.2 (N^+^CH_2_CH_2_CH_2 _ (CH_2_)_12_CH_2_CH_2_CH_3_), 23.0 (N^+^CH_2_CH_2_CH_2_(CH_2_)_12_CH_2_CH_2_CH_3_), 22.6 (N^+^CH_2_CH_2_CH_2_(CH_2_)_12_CH_2_ CH_2_CH_3_), 18.3 (CH_2_=C(CH_3_)), 14.1 (N^+^CH_2_CH_2_CH_2_(CH_2_)_12_CH_2_CH_2_CH_3_).

### 3.3. Minimum Inhibitory Concentration Determination (MIC)

The *in vitro* susceptibility tests were performed using broth microdilution. *S. mutans* Ingbritt was cultured overnight at +37 °C in Brain Heart Infusion medium (BHI; Difco, Detroit, MI, USA). In the morning *S. mutans* was transferred into fresh medium, the cells were cultured until the logarithmic phase, washed once with phosphate-buffered saline (10 min, 8,000 × *g*) and suspended in fresh BHI to an absorbance corresponding a cell density of 5 × 10^5^ CFU/mL. The monomers to be tested were dissolved in prewarmed BHI. Chlorhexidine (20%, Yliopiston apteekki, Helsinki, Finland) was diluted with water before used in the experiments. The cell solutions were combined with the monomer solutions so that in the reaction mixture (200 µL) the final cell concentration was 10^5^ and the final monomer concentrations ranged from 0.5 µg/mL to 100 µg/mL. After 24 h incubation at +37 °C the 96-well plate was shaken and the absorbance was measured (A_550_). The MIC value was the lowest monomer concentration in which no growth was detected. All experiments were performed with triplicates and repeated at least once. Purity of the organism was checked by taking samples of the cultures at all stages and culturing them on blood agar (Orion Diagnostica, Espoo, Finland). 

## 4. Conclusions

A series of polymeric iodine quaternary ammoniums salts with different alkyl chain lengths were synthesized by the reaction of dimethylaminoethyl methacrylate (DMAEMA) with different kinds of alkyl iodides, and their structures were characterized by FT-IR, ^1^H-NMR, and ^13^C-NMR analysis. In minimum inhibitory concentration determination, **C10** to **C18** showed significant antibacterial activity. The antibacterial activity increased with increasing alkyl side chain length of these monomers from five to 16, then decreased when the alkyl chain length increased to 18.

## References

[1] Skjörland K.K.R. (1973). Plaque accumulation on different dental filling materials. Scand. J. Dent. Res..

[2] Skjörland K.K.R. (1976). Bacterial accumulation on silicate and composite materials. J. Biol. Buccale.

[3] Zalkind M.M., Keisar O., Ever-Hadani P., Grinberg R., Sela M.N. (1998). Accumulation of *Streptococcus mutans* on light-cured composites and amalgam: An *in vitro* study. J. Esthet. Dent..

[4] Beyth N., Domb A.J., Weiss E. (2007). An *in vitro* quantitative antibacterial analysis of amalgam and composite resins. J. Dent..

[5] Weitman R.T., Eames W.B. (1975). Plaque accumulation on composite surface after various finishing procedure. J. Am. Dent. Assoc..

[6] Skjörland K.K.R., Sönju T. (1982). Effect of sucrose rinse on bacterial colonization on amalgam and composite. Acta Odontol. Scand..

[7] Günyakti N., Gür G., Misirligil A. (1990). *In vivo* adhesion of *Streptococcus mutans* on amalgam and composite restorative materials. Ankara Univ. Hekim. Fak. Derg..

[8] Auschill T.M., Arweiler N.B., Brecx M., Reich E., Sculean A., Netuschil L. (2002). The effect of dental restorative materials on dental biofilm. Eur. J. Oral Sci..

[9] Burk F.J., Crisp R.J., Bell T.J., Healy A., Mark B., McBirnie R., Osborne-Smith K.L. (2001). One-year retrospective clinical evaluation of hyrid composite restoration placed in United Kingdom general practices. Quintessence Int..

[10] Kenawy E.R., Worley S.D., Broughton R. (2007). The chemistry and applications of antimicrobial polymers: A state-of-art review. Biomacromolecules.

[11] Moran J., Addy M., Jackson R., Newcombe R.G. (2000). Comparative effects of quaternary ammonium mouthrinses on 4-day plaque regrowth. J. Clin. Periodontol..

[12] Simoncic B., Tomsic B. (2010). Structure of novel antimicrobial agents for textiles—A review. Text. Res. J..

[13] Imazato S., Torii M., Tsuchitani Y., McCabe J.F., Russell R.R.B. (1994). Incorporation of bacterial inhibitor into resin composite. J. Dent. Res..

[14] Imazato S., Kinomoto Y., Tarumi H., Torii M., Russell R.R.B., McCabe J.F. (1997). Incorporation of antibacterial monomer MDPB in dentin primer. J. Dent. Res..

[15] Imazato S., Torii Y., Takatsuka T., Inoue K., Ebi N., Ebisu S. (2001). Bactericidal effect of dentin primer containing antibacterial monomer methacryloyloxydodecylpyridinium bromide (MDPB) against bacteria in human carious dentin. J. Oral Rehabil..

[16] Imazato S., Ehara A., Totii M., Ebisu S. (1998). Antibacterial activity of dentine primer containing MDPB after curing. J. Dent..

[17] Ebi N., Imazato S., Noiri Y., Ebisu S. (2001). Inhibitory effects of resin composite containing bactericide-immobilized filler on plaque accumulation. Dent. Mater..

[18] Tanzer J.M., Livingston J., Thompson A.M. (2001). The microbiology of primary dental caries in humans. J. Dent. Educ..

[19] Järvinen H., Tenovuo J., Houvinen P. (1993). *In*
*vitro* susceptibility of *Streptococcus mutans* to chlorhexidine and six other antimicrobial agents. Antimicrob. Agents Chemother..

[20] Lu G., Wu D., Fu R. (2007). Studies on the synthesis and antibacterial activities of polymeric quaternary ammonium salts from dimethylaminoethyl methacryalte. React. Funct. Polym..

[21] Xie D., Weng Y., Guo X., Zhao J., Gregory R.L., Zheng C. (2011). Preparation and evaluation of a glass-ionomer cement with antibacterial functions. Dent. Mater..

[22] Rawlinson L.A., Ryan S.M., Mantovani G., Syrett J.A., Haddleton D.M., Brayden D.J. (2010). Antibacterial effects of poly(2-(dimethyamino ethyl)methacrylate) against selected gram-positive annnd gram-negative bacteria. Biomacromolecules.

[23] Thebault P., Taffin de Glvenchy E., Levy R., Vandenberghe Y., Guittard F., Geribaldi S. (2009). Preparation and antimicrobial behaviour of quaternary ammonium thiol derivatives able to be grafted on metal surfaces. Eur. J. Med. Chem..

[24] Balgavy P., Devinsky F. (1996). Cut-off effects in biological activities of surfactants. Adv. Colloid Interface. Sci..

[25] Caillier L., de Givenchy E.T., Levy R., Vandenberghe Y., Geribaldi S., Guittard F. (2009). Synthesis and antimicrobial properties of polymerizable quaternary ammoniums. Eur. J. Med. Chem..

